# Stroma, Stress and Mitotic Control in Prostate Cancer: Evaluating Periostin, MAPK8IP2, CDCA3 and NCAPG2 Within an Evidence Gap Framework

**DOI:** 10.3390/biology15212026

**Published:** 2026-11-07

**Authors:** Virginia Alonso-Aguirre, Alberto Sánchez-Espinosa, José García-Rodríguez, Antonio David Lázaro-Sánchez, Ginés Luengo-Gil

**Affiliations:** 1 Radiation Oncology Department, Hospital Universitario Santa Lucía, C/Mezquita, s/n, Paraje Los Arcos, 30202 Cartagena, Spain; 2 Doctoral Programme in Health Sciences, Universidad Católica San Antonio de Murcia (UCAM), Campus de los Jerónimos, Guadalupe, 30107 Murcia, Spain; 3 Group of Molecular Pathology and Pharmacogenetics, Medical Oncology and Pathology Departments, Instituto Murciano de Investigación Biosanitaria (IMIB), Hospital General Universitario Santa Lucía, 30202 Cartagena, Spain; 4 Medical Oncology Department, Hospital Clínico Universitario Virgen de la Arrixaca, Ctra. Madrid-Cartagena, s/n, 30120, El Palmar, 30120 Murcia, Spain

**Keywords:** prostate cancer, periostin, MAPK8IP2, CDCA3, NCAPG2, radiotherapy, biomarkers

## Abstract

Risk stratification in localised prostate cancer still relies mainly on clinical, pathological and genomic variables, whereas readily deployable tissue protein biomarkers remain insufficiently validated, particularly in patients treated with radiotherapy. This narrative review examines periostin (POSTN), mitogen-activated protein kinase 8 interacting protein 2 (MAPK8IP2/JIP2), cell division cycle-associated protein 3 (CDCA3) and non-SMC condensin II complex subunit G2 (NCAPG2) as illustrative candidates within a hypothesis-generating framework of potentially complementary biological processes. The review links the available evidence on these candidates to testable radiobiological hypotheses and specific experimental and clinical validation strategies. We searched PubMed on 25 October 2026 for publications dated from 1 January 2021 through 14 July 2026. We also included selected earlier publications. POSTN had the relatively strongest prostate-cancer tissue-level rationale, with stromal expression associated with adverse features and recurrence-related or metastatic biology. MAPK8IP2/JIP2 was associated with stress signalling, androgen-receptor-related states, and adverse features, but the evidence was mainly retrospective, transcriptomic, and experimental. CDCA3 was linked to cell-cycle and checkpoint regulation, whereas NCAPG2 was associated with chromosome organisation, malignancy, and STAT3/c-MYC-related stemness. However, no study identified in the literature reviewed demonstrated radiotherapy-specific predictive value, fractionation sensitivity, or androgen-deprivation benefit for any candidate. Future studies should use standardised compartment-specific immunohistochemistry, spatial assessment, patient-level model development, independent validation, comparison with clinical and genomic classifiers, and prespecified biomarker-by-treatment interaction analyses.

## Simple Summary

Choosing treatment for prostate cancer that has not spread beyond the prostate requires reliable ways to estimate the risk of the disease returning. Proteins measured in tumour tissue could complement existing assessments, but their usefulness remains uncertain, especially for patients receiving radiation treatment. This review examines research on four proteins involved in interactions between cancer and surrounding tissue, responses to stress within cells, and cell growth and division. The strongest evidence concerns periostin, a protein found in the tissue surrounding tumours, which has been linked to more aggressive disease and the return of cancer after treatment. Evidence for the other proteins is less established, although their functions suggest that they deserve further investigation. None of the four has been shown to identify which patients benefit more from radiation treatment. We outline how laboratory research and carefully designed patient studies could test these possibilities, using consistent methods to measure the proteins and confirming results in independent patient groups. This work provides a foundation for research that could eventually improve treatment decisions, while emphasising that these proteins are not yet ready to guide routine care.

## 1. Introduction

Prostate cancer (PCa) encompasses a wide biological spectrum, from indolent organ-confined tumours to rapidly recurrent, metastatic and treatment-resistant disease [1,2]. Contemporary management integrates prostate-specific antigen, clinical stage, International Society of Urological Pathology grade group (ISUP) [3], imaging and selected genomic classifiers, including integrated clinical-genomic risk systems [4]. These variables have improved risk stratification, yet substantial outcome heterogeneity remains within conventional risk groups, especially when the clinically relevant endpoint is long-term biochemical recurrence or metastatic progression after definitive treatment [5,6] (Figure 1).

Molecular classifiers add prognostic information and are increasingly studied in radiotherapy decision-making, but cost, tissue requirements, platform dependence and limited access can constrain their routine use [7,8].

Beyond conventional clinicopathological variables and established genomic classifiers, molecular stratification in PCa also encompasses genomic instability, candidate aneuploidy-associated driver programmes, treatment-related transcriptional signatures, and integrated tumour-microenvironment profiles. An integrative multi-omic analysis identified 11 candidate aneuploidy-associated driver genes and linked them to tumour progression, metastasis, and immune-microenvironmental features [9]. In a separate transcriptomic study, nine genes were associated with docetaxel-related features and immune infiltration, with ITGA2 emerging as a candidate with prognostic implications [10]. More recent work in metastatic castration-resistant prostate cancer has emphasised the coexistence of androgen-receptor-dependent and androgen-receptor-independent resistance mechanisms, supporting biomarker-guided patient selection and rational combination strategies [11].

Within this evolving landscape, a useful biomarker framework should define the biological state being measured, determine whether the marker provides information beyond established variables, and distinguish prognostic from predictive value [12]. Prognostic biomarkers are associated with outcome within a defined clinical context, whereas predictive biomarkers identify differential benefit from a particular intervention and therefore require a formal treatment-by-biomarker interaction analysis [13].

Against this background, we examine four candidate proteins—POSTN, MAPK8IP2/JIP2, CDCA3, and NCAPG2. They were selected through an exploratory, hypothesis-driven process rather than through a formal or exhaustive ranking of prostate-cancer biomarkers. An initial PubMed search focused on novel or relatively undercharacterised protein biomarkers associated with PCa prognosis, tumour progression, treatment adaptation, or stromal and cellular biology. Candidate proteins were subsequently cross-referenced with publicly available protein-expression and pathology resources, including The Human Protein Atlas and its Pathology Atlas, to assess prostate-tissue expression, protein localisation, potential immunohistochemical detectability, and available clinical associations [14].

The selection framework was guided by four considerations: relevance to human PCa biology; a plausible and biologically distinct role in tumour progression or treatment adaptation; potential measurability in tissue using compartment-aware immunohistochemistry; and representation of biological dimensions not reducible to a single proliferation measure. These criteria were refined during the exploratory literature synthesis and were not prospectively registered or applied as a formal candidate-selection algorithm before the initial search.

The selected candidates were intended to represent potentially complementary biological dimensions and tissue compartments: POSTN is associated with reactive stroma and extracellular-matrix remodelling [15], MAPK8IP2/JIP2 with stress-signalling organisation and an androgen-receptor-associated context [16], CDCA3 with mitotic entry and checkpoint regulation [17], and NCAPG2 with condensin-associated chromosome organisation and stemness-related programmes [18]. Biomarkers not included in this framework were not considered biologically uninformative or inferior; rather, they were outside the scope of this focused model because they did not fit the intended biological dimensions, were potentially redundant with the selected candidates, or lacked sufficiently direct prostate-cancer tissue or mechanistic evidence in the exploratory search.

This four-protein candidate set is therefore intended as a focused, hypothesis-generating framework rather than as an exhaustive or clinically validated panel. Whether these protein measurements provide information independent of, or incremental to, existing genomic classifiers remains unknown. Current classifiers may capture overlapping biological processes, including proliferation, tumour-cell state, and other molecular features. The potential contribution of the proposed framework therefore requires direct same-cohort comparison using nested clinical, genomic, protein-level, and integrated models.

## 2. Search Strategy and Scope

A focused narrative literature search was conducted exclusively in PubMed/MEDLINE. We ran the searches on 25 October 2026. For the core query and the four marker-specific queries, we restricted publication dates to the period from 1 January 2021 through 14 July 2026. SANRA was used as a critical appraisal framework for the narrative review [19], whereas the search methods were documented according to relevant principles of the PRISMA-S extension, including the search platform, search date, complete search syntax, applied limits, and record counts [20].

The core PubMed/MEDLINE query was: ((“Prostatic Neoplasms”[Mesh] OR “prostate cancer”[Title/Abstract] OR “prostatic neoplasms”[Title/Abstract] OR “prostate adenocarcinoma”[Title/Abstract]) AND (POSTN[Title/Abstract] OR periostin[Title/Abstract] OR MAPK8IP2[Title/Abstract] OR JIP2[Title/Abstract] OR CDCA3[Title/Abstract] OR “TOME-1”[Title/Abstract] OR TOME1[Title/Abstract] OR NCAPG2[Title/Abstract] OR “condensin II”[Title/Abstract]) AND (“2021/01/01”[Date—Publication]: “2026/07/14”[Date—Publication])).

This query retrieved 24 records. Four marker-specific supplementary PubMed queries used the same prostate-cancer and publication-date blocks and combined each candidate with prespecified terms related to radiotherapy, radiation, radioresistance, biochemical recurrence, prognosis, immunohistochemistry, androgen-receptor signalling, cancer stem cells, extracellular matrix, JNK, p38, STAT3, MYC, WEE1, and cell cycle. These queries retrieved 8 records for POSTN/periostin, 3 for MAPK8IP2/JIP2, 3 for CDCA3/TOME-1, and 5 for NCAPG2/condensin II. The complete syntax of the four supplementary queries is provided in the Supplementary Methods.

No systematic searches were conducted in Embase, Scopus, Web of Science Core Collection, the Cochrane Library, Google Scholar, ClinicalTrials.gov, the World Health Organization International Clinical Trials Registry Platform, preprint servers, conference-proceeding databases, or other grey-literature sources. Non-PubMed records were therefore not individually screened or excluded because these sources were outside the prespecified information-source framework.

Across the core and supplementary PubMed queries, 43 query-level retrievals were recorded before accounting for overlap. Because the supplementary queries overlapped with the core query, 19 overlapping retrievals were removed, leaving 24 unique records for title and abstract screening. We excluded three records at title and abstract screening and assessed the remaining 21 articles in full. Nine articles concerned postoperative or metastatic disease and provided no tissue biomarker, mechanistic or radiotherapy evidence relevant to the questions addressed in this review. We retained 12 publications from the date-restricted search.

We identified four earlier publications through targeted PubMed searches without a lower publication-date limit and through citation tracking. Together with the 12 publications retained from the date-restricted search, these brought the total to 16. The Supplementary Methods identify the four earlier publications and describe their contribution to the review.

Evidence from other tumour types was used only to support general mechanistic principles when prostate-cancer-specific data were unavailable and is explicitly identified as indirect. SANRA was not used as a substitute for transparent searching, eligibility criteria, screening, or evidence selection. Because the aim was a critical narrative synthesis, no meta-analysis or formal study-level risk-of-bias scoring was performed. Conclusions were weighted according to biological validation, replication across cohorts, tissue-level confirmation, methodological consistency, and directness to prostate cancer and radiotherapy.

Because the available studies differed substantially in disease stage, treatment exposure, tissue source, assay type, and experimental model, they were not considered interchangeable. Evidence from untreated or treatment-naïve localised prostate cancer was distinguished from evidence obtained after surgery, radiotherapy, androgen-deprivation therapy, chemotherapy, or in recurrent, metastatic, or metastatic castration-resistant disease. Cell-line, xenograft, organoid, transcriptomic, proteomic, and immunohistochemical studies were also interpreted as distinct evidence categories.

Potential confounding factors included prior treatment, androgen-deprivation exposure, radiotherapy dose and fractionation, tumour burden, castration-resistant status, sampling site, multifocality, and treatment selection. Associations observed in retrospective treated cohorts were therefore interpreted as prognostic or hypothesis-generating unless supported by an appropriate comparative design and a prespecified biomarker-by-treatment interaction. Findings from advanced or previously treated disease were not extrapolated directly to untreated localised prostate cancer.

## 3. A Conceptual Framework of Candidate Biological Dimensions

The tumour microenvironment in prostate cancer (PCa) is a dynamic context in which malignant epithelial cells interact with stromal, immune, and extracellular-matrix components [21,22]. Tumour-cell genetic and epigenetic alterations, together with changes in stromal composition and state, may influence intercellular communication and contribute to heterogeneity. Conversely, stromal programmes may be associated with tumour aggressiveness, cellular plasticity, and metastatic potential [23]. These relationships are context-dependent and do not imply that individual tumour alterations uniformly determine stromal behaviour.

Within this context, POSTN, MAPK8IP2/JIP2, CDCA3, and NCAPG2 were selected as candidate markers representing potentially complementary biological dimensions rather than as components of an established interconnected system. POSTN is a candidate marker of reactive stroma and extracellular-matrix remodelling. MAPK8IP2/JIP2 is associated with stress-signalling organisation and androgen-receptor-related states in selected prostate-cancer models. CDCA3 is associated with mitotic entry and checkpoint regulation. NCAPG2 is involved in condensin-II-related chromosome organisation and has been linked experimentally to a STAT3/c-MYC-associated stemness programme.

These candidates therefore span different biological contexts: the extracellular tumour–stroma interface, stress adaptation, cell-cycle and checkpoint regulation, and chromosome organisation or tumour-cell state. This organisation is useful for generating testable hypotheses, but it does not demonstrate direct molecular interaction, coordinated regulation, spatial colocalisation, or a shared adaptive pathway among the four proteins.

The framework may also help formulate clinically relevant questions. For example, stromal POSTN expression could provide information that differs from tumour-cell proliferation, whereas CDCA3 or NCAPG2 expression could identify tumour-cell states not fully represented by stromal measurements. MAPK8IP2/JIP2 may capture stress- or lineage-associated biology that overlaps partially with, but is not necessarily independent of, androgen-receptor activity or established proliferation measures. These possibilities remain untested and should not be interpreted as demonstrated incremental value.

The model should therefore be regarded as hypothesis-generating. The central translational question is not whether these proteins can be connected conceptually, but whether compartment-specific protein measurements are reproducible and provide prognostic or predictive information beyond clinical variables, established proliferation markers, and validated genomic classifiers. Direct functional connectivity, spatial co-occurrence, and treatment-specific complementarity require dedicated experimental and clinical validation.

### Spatial Compartment Is Part of the Biomarker Definition

Most available evidence for MAPK8IP2, CDCA3, and NCAPG2 is derived from retrospective transcriptomic datasets. These data are valuable for identifying candidate biological programmes but should not be interpreted as direct measurements of protein abundance, cellular origin, or spatial distribution. In localised prostate cancer, transcript–protein concordance is incomplete, and post-transcriptional regulation, protein stability, translational efficiency, and tissue admixture may substantially alter the relationship between mRNA and protein levels. This issue is particularly important for the present candidate set because POSTN is hypothesised to be enriched in reactive stromal compartments, whereas the dominant cellular and subcellular localisation of MAPK8IP2, CDCA3, and NCAPG2 requires direct protein-level confirmation.

Accordingly, transcriptomic associations should be described as candidate-gene or pathway-level evidence, rather than as definitive evidence that the corresponding proteins are overexpressed in the same cells or tissue regions. Protein-level validation should include analytically validated antibodies, orthogonal confirmation where feasible, compartment-specific scoring, blinded quantitative image analysis, and assessment in independent pretreatment biopsy cohorts. Multiplex immunofluorescence, imaging mass cytometry, or other spatial-profiling approaches may help determine whether transcript-derived signals correspond to tumour, stromal, immune, or mixed cellular populations. Until such validation is available, protein-level and radiobiological interpretations remain hypothesis-generating.

Spatial analysis may also permit evaluation of cross-compartment relationships, such as the proximity of POSTN-rich stroma to tumour regions expressing MAPK8IP2, CDCA3, or NCAPG2. These patterns could identify spatially defined tumour–stroma configurations, but their reproducibility and biological significance remain unproven. Digital pathology and multiplex immunohistochemistry may help quantify such relationships [24,25]. A practical development strategy would retain separate marker- and compartment-specific measurements, prespecify any interaction terms, and derive a composite score only in a defined development cohort. The complete model should then be locked before independent external validation.

These biological interpretations remain provisional pending spatial, protein-level, and clinical validation. The compartment-specific measurements are summarised in Table 1 and illustrated in Figure 2.

These categories are narrative summaries, not formal GRADE assessments. Moderate denotes convergent findings across studies and assay types, including tissue-level evidence, with incomplete independent clinical validation. Limited–moderate denotes supportive functional, transcriptomic or tissue findings with restricted replication and insufficient protein-level clinical validation. Limited denotes a sparse or predominantly single-study evidence base with little independent clinical confirmation. Mechanistic support, protein-level prognostic validation and radiotherapy-specific predictive evidence are distinct dimensions; support for one does not establish the others. Repeated analyses of the same patient cohort do not constitute independent clinical replication.

## 4. Periostin: Reactive Stroma, Matrix Remodelling and Metastatic Potential

POSTN is a secreted extracellular-matrix (ECM) protein that binds integrins and contributes to collagen organisation and matrix remodelling, thereby influencing adhesion, migration, tissue stiffness, and paracrine signalling. In cancer, POSTN is produced predominantly by cancer-associated fibroblasts (CAFs), although epithelial cells and osteoblasts may also contribute. This source heterogeneity is particularly relevant in prostate cancer (PCa), where reactive stromal responses accompany disease progression and bone is the dominant metastatic site [26,27].

Across transcriptomic and clinical datasets, the biological interpretation of POSTN depends on disease state and cellular compartment. Stromal POSTN has been associated with aggressive primary PCa, whereas epithelial expression has been described more frequently in advanced or metastatic contexts [28]. These associations should not be assumed to represent a uniform disease-wide POSTN state.

Stromal POSTN has shown protein-level prognostic associations in selected tissue studies [29], but the strength and generalisability of this evidence depend on antibody performance, stromal compartment definition, sampling site, disease state, and treatment context. POSTN may be measurable in routine formalin-fixed tissue, provided that the assay is analytically validated and stromal and epithelial staining are scored separately. Proteomic studies have also included POSTN in signatures distinguishing aggressive from indolent disease [30], although this does not establish POSTN as a stand-alone clinical assay.

Mechanistic studies have implicated tumour- and osteoblast-derived POSTN in reciprocal signalling within the bone microenvironment, with associations with niche remodelling, proliferation, and invasion [31]. These findings support a possible role for POSTN in metastatic-niche biology, particularly in bone, but their relevance to untreated localised disease remains uncertain. Associations between high POSTN and shorter recurrence-free intervals should therefore be interpreted within the disease and treatment context in which they were observed.

Stromal characteristics may also contribute to imaging–pathology discordance. High stromal POSTN has been associated with false-positive magnetic resonance imaging assessments of extraprostatic extension, suggesting that stromal remodelling may influence the relationship between imaging findings and histopathology [32]. This observation does not establish POSTN as an imaging biomarker or as a predictor of radiotherapy response.

In aggressive PCa models, POSTN expression has been associated with intense stromal remodelling, reduced androgen-receptor expression at the invasive front, dedifferentiated features, neuroendocrine-associated programmes, and metastatic behaviour [23]. These findings support biological plausibility but do not establish that POSTN directly causes lineage plasticity or metastatic dissemination.

Proposed links between POSTN and treatment resistance include integrin–FAK–PI3K/AKT signalling, ECM stiffness, CAF-derived inflammatory signals, and hypoxia. Much of this rationale is indirect or derived from broader tumour-microenvironment literature [33]. Although radiotherapy can alter stromal composition and fibroblast activity, no study has demonstrated that POSTN mediates a radiotherapy-specific resistant niche in prostate cancer.

Differences among POSTN studies may reflect several non-mutually exclusive factors, including disease stage, tumour versus stromal compartment, fibroblast and osteoblast composition, multifocal sampling, prior treatment, assay characteristics, and cohort selection. Biological heterogeneity is therefore a plausible explanation, but sampling error and analytical variation cannot be excluded. Comparisons between primary, recurrent, and metastatic disease should consequently be interpreted cautiously.

Within the literature reviewed, no study demonstrated that pretreatment POSTN predicts radiotherapy benefit, radioresistance, fractionation sensitivity, or benefit from androgen-deprivation therapy. POSTN should therefore be considered a stromal prognostic candidate with mechanistic plausibility, not an established predictive biomarker.

Future studies should score stromal and epithelial POSTN separately in pretreatment biopsies and correlate these measurements with biochemical recurrence and metastatic outcomes after radiotherapy. Comparative analyses should test prespecified interactions with dose, fractionation, and androgen-deprivation therapy. Integrating hypoxia, matrix-remodelling, and integrin-signalling measurements may help determine whether POSTN is a functional mediator or a marker of broader stromal states.

## 5. MAPK8IP2/JIP2: A Stress-Signalling Scaffold in an Androgen-Receptor-Related Context

MAPK8IP2, also known as JIP2, is a scaffold protein associated with JNK/p38 mitogen-activated protein kinase (MAPK) stress-signalling pathways, which regulate cell migration, proliferation, differentiation, and intracellular transport [34]. Because JNK/p38 signalling can promote apoptosis after severe cellular damage or adaptive responses under chronic stress, MAPK8IP2 may influence the balance between these outcomes in a context-dependent manner [35]. The proposed signalling context is illustrated in Figure 3.

Three principal prostate-cancer studies currently inform the evidence base for MAPK8IP2/JIP2, although they address different biological questions and should not be considered independent clinical replication.

Zhenhao et al. described a proposed post-transcriptional circMAML3–miR-665–MAPK8IP2 circuit in which circMAML3 sequesters the tumour-suppressive miR-665 and increases MAPK8IP2 expression. Functional experiments associated this circuit with proliferation, migration, invasion and xenograft growth, identifying MAPK8IP2 as a downstream effector of the proposed regulatory pathway [36]. These findings provide mechanistic and functional evidence for a post-transcriptional MAPK8IP2 programme but do not establish a radiotherapy-specific mechanism.

Zeng et al. analysed public prostate-cancer datasets and performed MAPK8IP2 perturbation experiments in prostate-cancer cell lines. Higher MAPK8IP2 transcript expression was associated with adverse clinicopathological features and poorer outcomes, while MAPK8IP2 suppression reduced proliferation, migration, and invasion [37]. These findings support a transcriptomic and functional association with aggressive prostate-cancer biology. However, the analyses were retrospective, the functional experiments were performed in cell lines, and independent protein-level and radiotherapy validation were not provided.

Huang et al. evaluated MAPK8IP2 in clinical datasets and examined its relationship with androgen-receptor (AR) signalling. Androgen stimulation increased MAPK8IP2 expression; AR-pathway inhibition reduced expression in LNCaP, but not C4-2B or 22RV1 cells [16]. These findings support an AR-associated and context-dependent relationship, but they do not establish that MAPK8IP2 defines an AR-dependent phenotype. The relationship may vary according to treatment history, lineage state, and the degree of residual AR activity. Huang et al. also observed cytoplasmic MAPK8IP2 staining in a tissue microarray, without establishing protein-level prognostic validity [16].

Across these studies, MAPK8IP2 expression has also been associated with DNA-methylation patterns, selected mutational contexts, and immune-related features. These observations are hypothesis-generating and do not establish whether MAPK8IP2 is an independent driver, a downstream effector, or a surrogate of broader transcriptional, epigenetic, lineage, or stress-adaptation programmes.

MAPK8IP2 is therefore a plausible candidate for protein-level and radiobiological investigation, but its relevance to radiation response remains unproven. Ionising radiation activates stress-responsive MAPK pathways in general, but no prostate-cancer study has yet evaluated clonogenic survival after irradiation, radiation-induced DNA-damage repair, in vivo radiotherapy response, or combined MAPK8IP2 perturbation and AR inhibition under clinically relevant fractionation. Accordingly, the potential effect of MAPK8IP2 on radiation-induced apoptosis or adaptive recovery remains an untested hypothesis.

Current evidence is predominantly transcriptomic and functional and does not establish reproducible spatial protein abundance in localised prostate cancer. Transcript abundance should not be assumed to predict protein staining, particularly across hormone-sensitive, castration-resistant, AR-low, and AR-null states. Future studies should validate antibody specificity and quantify MAPK8IP2 in defined tumour-cell compartments, with parallel assessment of AR activity, phospho-JNK/p38 signalling, and relevant stromal and immune markers. These studies should determine whether MAPK8IP2 provides independent protein-level information or primarily reflects broader lineage and stress-related programmes.

## 6. CDCA3: Checkpoint Regulation, Proliferative Biology and Untested Radiobiological Relevance

CDCA3 (TOME-1) is a cell-cycle regulator associated with mitotic entry and WEE1-related checkpoint control [38]. In prostate-cancer models, CDCA3 suppression was accompanied by NF-κB/cyclin D1 inactivation, p21 accumulation, cell-cycle arrest, and apoptosis [17] (Figure 4).

These findings support a functional association between CDCA3 and checkpoint/proliferative programmes, but they do not demonstrate direct CDCA3–p21 regulation or establish a radiotherapy-specific mechanism.

CDCA family genes consistently appear among transcriptomic signatures of adverse pathological features and poor clinical outcomes [39]. However, family-level analyses do not establish the independent biological contribution of CDCA3 itself [39]. High CDCA3 transcript levels have been associated with proliferative and adverse clinical programmes in retrospective datasets, but it remains unknown whether these associations correspond to increased CDCA3 protein abundance, reproducible compartment-specific staining, or a phenotype beyond generic cell-cycle activity.

Functional studies provide additional mechanistic support. Silencing CDCA3 in prostate-cancer cell lines was associated with G0/G1 arrest, NF-κB/cyclin D1 inactivation, p21 accumulation, increased apoptosis, and reduced tumour-cell growth and progression. These concurrent changes support a pathway-associated response but do not demonstrate a direct CDCA3–p21 interaction [17].

Gu et al. analysed CDCA3 mRNA in TCGA and protein expression in a separate IHC series comprising 60 prostate cancers and 20 normal samples. High transcript expression was associated with a shorter progression-free interval in univariable Cox analysis (*n* = 497; HR 2.237, 95% CI 1.447–3.458), without establishing prognostic independence. The reported AUC of 0.857 concerned diagnostic discrimination between cancer and normal tissue, not survival prediction. In the IHC series, protein expression was associated with PSA, T stage and Gleason score. These tissue findings do not validate protein-level survival prediction [40].

Accordingly, current evidence supports an association between CDCA3 and proliferative or adverse disease features, but does not establish CDCA3 protein as an independent prognostic biomarker. Transcript-level findings should not be treated as surrogates for protein expression without direct validation. Protein-level studies should evaluate CDCA3 alongside Ki-67, mitotic count, and established cell-cycle signatures in the same tissue cohort, while reporting subcellular localisation, staining intensity, and the proportion of positive tumour cells separately.

Established multigene cell-cycle signatures provide an important benchmark for evaluating CDCA3. The 31-gene cell-cycle progression signature developed by Cuzick et al. provided prognostic information beyond standard clinical variables in prostate cancer, but this RNA-based signature does not establish that CDCA3 protein adds independent information beyond established measures of proliferation [41].

CDCA3 biology provides a plausible but untested radiobiological hypothesis. Accelerated cell-cycle entry could increase mitotic vulnerability, whereas altered checkpoint use or survival-associated signalling could facilitate recovery after irradiation [42]. Within the literature reviewed, no study demonstrated that CDCA3 modifies clonogenic survival, repair of radiation-induced DNA damage, mitotic catastrophe, or radiotherapy benefit in prostate cancer.

Definitive evaluation requires CDCA3 perturbation under clinically relevant irradiation conditions, with assessment of clonogenic survival, cell-cycle redistribution, persistent DNA damage, and mitotic catastrophe. Combinations with WEE1 or androgen-receptor inhibition may help define the relevant checkpoint context.

Clinically, CDCA3 should be tested alongside established proliferation markers and within comparative radiotherapy datasets when a predictive claim is intended. Until radiation-specific interactions are demonstrated, CDCA3 should be regarded as a candidate marker of proliferative and checkpoint dysregulation rather than a validated radioresistance biomarker.

## 7. NCAPG2: Condensin II, Chromosomal Organisation and Tumour Stemness

NCAPG2 is a condensin II regulatory subunit, which maintains chromatid structure and ensures proper chromosome condensation, organisation and segregation. Disruption of condensin II can lead to aneuploidy and genomic instability [43,44].

In prostate cancer, NCAPG2 mRNA expression has been associated with proliferative, invasive, and stemness-related programmes in available datasets. Experimental studies have implicated STAT3/c-MYC signalling in the malignant and stemness-related effects of NCAPG2, but transcriptomic association alone does not establish increased protein abundance, nuclear condensin-II activity, or a spatially restricted tumour-cell state [18].

Because NCAPG2 may be detected in both nuclear and cytoplasmic compartments, protein-level studies should report nuclear and cytoplasmic staining separately. Nuclear-specific quantification, ideally combined with markers of tumour identity, proliferation, and stemness, is required before assigning condensin-related biological meaning to the signal.

The available non-irradiated evidence linking NCAPG2 to chromosome organisation, STAT3/c-MYC signalling, malignancy, and stemness-related phenotypes provides a rationale for investigating its potential relevance to radiotherapy response, but does not establish a radiation-specific mechanism. Within the literature reviewed, the available clinical and functional evidence was derived principally from one multi-platform prostate-cancer study, without independent protein-level replication. NCAPG2–MYC co-expression was also examined in GSE116918 [18], a cohort treated with radiotherapy and androgen-deprivation therapy. This analysis does not establish an association with radiotherapy outcome or a biomarker-by-treatment interaction. No direct irradiation experiments were identified.

A high-NCAPG2 expression state could theoretically be associated with increased vulnerability to radiation-induced chromosome damage or, alternatively, with enhanced post-treatment recovery and tumour-cell persistence. The direction of this relationship remains unknown. NCAPG2 is therefore a candidate marker of tumour-cell state whose possible relationship with radiosensitivity, radioresistance, and recurrence remains hypothesis-generating [45,46,47].

Whether NCAPG2 provides information beyond Ki-67, mitotic count, or established cell-cycle signatures has not been demonstrated. This question requires head-to-head comparison in the same tissue cohort, adjustment for established proliferation measures, and independent validation.

Mechanistic clarification will require dose–response studies, clonogenic assays, persistent DNA-damage measurements, chromosome-aberration analysis, rescue experiments, and assessment of delayed clonogenic regrowth after clinically relevant fractionated radiation. Re-expression of separation-of-function NCAPG2 mutants after knockdown could help determine whether post-radiation survival depends on chromosomal architecture, STAT3 binding, or both. Future clinical studies should validate NCAPG2 in independent tissue cohorts and diagnostic biopsies and compare it directly with Ki-67 and established cell-cycle genomic signatures. Table 2 provides a quantitative and methodological evidence map.

## 8. Convergence on Progression and Potential Radiation Response

The radiobiological rationale for the four candidates should be interpreted according to the directness of the available evidence. Four evidence categories are distinguished: direct prostate-cancer evidence without radiotherapy, direct evidence from irradiated prostate-cancer models or radiotherapy-treated cohorts, indirect evidence from general radiobiology or other tumour types, and untested radiobiological hypotheses.

For POSTN, current prostate-cancer evidence concerns stromal remodelling, extracellular-matrix organisation, aggressive disease, recurrence-related phenotypes, and bone-metastatic biology, primarily in non-irradiated or advanced-disease settings. For MAPK8IP2/JIP2, evidence concerns stress-signalling organisation, androgen-receptor-associated states, and malignant cellular phenotypes. For CDCA3, evidence concerns mitotic entry, checkpoint regulation, proliferation, and adverse clinicopathological features. For NCAPG2, evidence concerns chromosome organisation, STAT3/c-MYC signalling, malignancy, and stemness-related phenotypes.

These associations were interpreted within their original disease and treatment contexts. Findings from advanced, previously treated, or non-irradiated disease were not assumed to represent the biology of pretreatment localised prostate cancer undergoing radiotherapy.

Within the literature reviewed, no study was identified showing that pretreatment expression of POSTN, MAPK8IP2/JIP2, CDCA3, or NCAPG2 modifies clonogenic survival after irradiation, repair of radiation-induced DNA damage, in vivo response to radiotherapy, or the relative benefit of dose, fractionation, or androgen-deprivation strategies. Evidence from other tumour types may support general principles—for example, the contribution of extracellular-matrix remodelling, stress responses, checkpoint control, or stemness to treatment resistance—but cannot establish the role of the corresponding protein in prostate-cancer radiotherapy.

The four proteins should therefore be interpreted as candidate biological dimensions rather than components of a validated molecular axis or radiotherapy-response panel. They may be relevant to recurrence and treatment response through stromal remodelling, stress adaptation, checkpoint dysregulation, chromosome organisation, and tumour-cell persistence. Biological convergence is plausible because radiotherapy simultaneously affects several of these processes, including DNA damage, oxidative stress, fibroblast activation, vascular remodelling, immune composition, and extracellular-matrix organisation [48,49,50]. However, shared exposure to radiation does not demonstrate that these proteins function as an integrated response network.

Table 3 separates direct prostate-cancer evidence from direct irradiation evidence, indirect evidence derived from general radiobiology or other tumour types, and hypotheses that remain untested. The potential predictive value of the four candidates therefore requires direct testing in clinically relevant irradiation experiments and through formal biomarker-by-treatment interaction analyses.

Evidence classification: “Direct prostate-cancer evidence” refers to studies performed in human prostate-cancer cohorts, prostate-cancer tissue, or prostate-cancer models without necessarily involving radiotherapy. Direct radiotherapy evidence refers to studies using irradiated prostate-cancer models or clinical cohorts treated with radiotherapy. Prognostic associations within such cohorts must be distinguished from predictive evidence, which requires an appropriate treatment comparison and a biomarker-by-treatment interaction analysis. “Indirect evidence” refers to general radiobiological evidence or studies in other tumour types and is not interpreted as proof of marker-specific effects in prostate cancer. “Potential hypothesis” denotes an untested mechanism and does not imply established radiosensitivity, radioresistance, prognostic validity, or clinical utility. Abbreviations: CDCA3, cell division cycle-associated protein 3; IHC, immunohistochemistry; JNK, c-Jun N-terminal kinase; PCa, prostate cancer; WEE1, Wee1 G2 checkpoint kinase.

Existing radiotherapy biomarker studies in prostate cancer highlight the critical distinction between prognostic and predictive biomarkers. In translational analyses of the randomised CHHiP trial [51] and in the prospective biomarker study by Pollack et al. [52], which was nested within a randomised hypofractionation trial, Ki-67 was associated with an increased risk of recurrence or biochemical/clinical disease failure, supporting its prognostic value. However, these studies did not demonstrate fraction-size sensitivity or a differential benefit from a specific radiotherapy schedule. Similarly, studies evaluating hypoxia- and angiogenesis-related markers such as osteopontin and GLUT1 found associations with biochemical relapse-free survival in radiotherapy-treated patients, although treatment-specific effects remained exploratory and required further validation [53].

These studies illustrate the clinical designs required to evaluate treatment-related biomarkers, but do not provide evidence supporting POSTN, MAPK8IP2/JIP2, CDCA3, or NCAPG2 as validated predictive markers of radiotherapy response.

Clinical validation should distinguish several questions. Prognostic validation after radiotherapy asks whether pretreatment biomarker expression is associated with outcome within a defined radiotherapy context and can initially be performed in a well-characterised radiotherapy-treated cohort. Prediction of fractionation benefit requires comparison of radiotherapy schedules. Prediction of androgen-deprivation therapy (ADT) benefit requires comparison of radiotherapy with versus without ADT or of different ADT durations. Prediction of radiotherapy benefit relative to another treatment requires a cohort containing both treatment strategies. Each predictive question requires a prespecified biomarker-by-treatment interaction rather than comparisons of outcomes within biomarker strata alone.

Treatment-by-biomarker interactions estimated from non-randomised comparisons are vulnerable to confounding by indication, treatment-selection bias, baseline imbalance, differential follow-up, and unmeasured factors influencing treatment assignment and outcome. Multivariable adjustment, propensity scores, weighting, or target-trial emulation may improve comparability but cannot recreate randomisation and depend on adequate measurement of confounders. Such analyses should therefore be considered exploratory unless supported by prespecified estimands and analyses, adequate adjustment, sensitivity analyses, and independent validation.

A staged translational programme should therefore proceed from analytical validation to clinical model development, patient-level internal validation, algorithm locking, independent external validation, and subsequent assessment of clinical utility and treatment-specific biomarker interactions. The operational details of composite-score development and tissue-level clinical validation are described in sections Designing a Composite Score and Section 9. Organoid models and patient-derived cultures could complement this pathway by enabling functional validation and radiation-sensitivity testing in systems that preserve tumour architecture [54,55].

### Designing a Composite Score

A future four-marker score should be regarded as a statistical hypothesis about incremental information, not as a presumed biological composite. Any apparent improvement in model performance would require confirmation that it is reproducible, clinically useful, and not merely attributable to correlated measures of tumour burden, proliferation, stromal content, or sampling heterogeneity.

The development cohort should use the patient as the unit of prediction while preserving the nested structure of measurements obtained from regions of interest, cores, lesions, and patients. Candidate patient-level summaries should be prespecified before outcome analysis. These may include an index-lesion score, a patient-level average across sampled lesions, the highest-risk lesion, and an explicit measure of inter-lesional heterogeneity. Resampling and data splitting should be performed at the patient level to prevent information leakage between cores or lesions from the same patient.

Model development should use continuous marker values whenever analytically reliable. Candidate structures may include a weighted linear score, a two-domain score separating stromal and tumour-cell measurements, a rule-based model identifying prespecified combinations, and a spatial model assessing colocalisation between POSTN-rich stroma and NCAPG2- or CDCA3-high tumour regions. These models are exploratory; no current evidence demonstrates that such combinations improve prediction or reflect a biologically integrated network.

Penalised regression and prespecified interactions may be used during model development to reduce overfitting, with model complexity constrained by the available sample size and number of outcome events. Patient-level bootstrap or nested cross-validation should be used for internal validation, estimation of optimism, and, where appropriate, model updating. The complete model—including normalisation, compartment assignment, patient-level aggregation, missing-core handling, coefficients, interaction terms, and thresholds—should be locked only after model development and internal validation have been completed.

The locked algorithm should subsequently undergo independent external validation without outcome-driven changes to its coefficients or thresholds. Any recalibration performed for a new population should be prespecified, reported separately from the primary validation, and not presented as confirmation of the original locked model. Performance should be assessed using calibration, discrimination, prediction error, and clinical utility, including incremental value over a prespecified clinical baseline model. These properties remain unknown for the proposed score and may vary according to the number of sampled lesions, the number of interpretable cores, and the degree of intraprostatic heterogeneity.

## 9. Immunohistochemical Implementation and Clinical Validation

Translational evaluation of the panel should begin with analytical validation. Whole-section tissue should be used where feasible to assess expected protein localisation, tissue architecture, staining range, and suitability of candidate clones. Antibody specificity should be supported by appropriate positive and negative controls and, where feasible, orthogonal confirmation or comparison with a validated assay. The analytical phase should also assess fixation, tumour content, block age, antigen-retrieval and antibody parameters, staining platform, digital-image segmentation, and interobserver or interlaboratory reproducibility. For multiplex assays, antibody clones, platform, scoring system, image-analysis workflow, and quality-control procedures should be reported explicitly.

POSTN should be evaluated primarily in stromal regions, with epithelial staining recorded separately. MAPK8IP2 and CDCA3 require direct confirmation of their dominant tumour-cell and subcellular localisation. NCAPG2 should be scored separately in nuclear and cytoplasmic compartments because these signals may represent different biological processes. The most reproducible scoring metric—intensity, percentage positivity, H-score, area-based measurement, or a spatial metric—should be selected before clinical outcome modelling.

Multifocality should be treated as a prespecified design feature. Each core should retain its patient, lesion, anatomical location, and targeted or systematic sampling status. The validation dataset should document the number of sampled lesions, number of cores per lesion, tumour length, tumour percentage, Grade Group, and interpretable stromal tissue.

Multiple cores may improve tissue representation but do not guarantee biological representativeness. Small cores may fail to capture invasive margins, reactive stroma, or spatially restricted CDCA3- or NCAPG2-high subclones. Tissue microarrays may facilitate laboratory standardisation but can underestimate lesion-level heterogeneity. Whole-section analysis or spatially annotated serial sections should therefore be included in an analytical substudy whenever feasible.

Patient-level aggregation should follow the prespecified strategy described in Designing a Composite Score section . Index-lesion scores, weighted averages across sampled lesions, highest-risk lesions, or hierarchical estimates may be compared in sensitivity analyses, but should not be selected solely because they produce the strongest association with outcome. Missing or non-interpretable cores should be recorded as missing and incorporated into the missing-data strategy rather than assigned a low biomarker value.

The primary clinical analysis should first evaluate prognostic associations after radiotherapy in pretreatment biopsy cohorts with defined follow-up and standardised endpoints. Models should adjust for clinical risk, Grade Group, prostate-specific antigen, tumour volume, magnetic resonance imaging findings, treatment variables, and relevant ADT exposure. Prespecified endpoints may include biochemical recurrence, metastasis-free survival, and prostate-cancer-specific mortality.

The panel should then be compared with realistic clinical and molecular benchmarks in the same patient cohort. Comparisons should include: (1) a clinical baseline model; (2) an appropriate commercial genomic classifier, where available and measured in the same cohort; (3) the clinical model plus the genomic classifier; (4) the clinical model plus the four-protein panel; and (5) an integrated clinical, genomic, and protein model. Identical endpoints and follow-up horizons should be used, with prespecified assessment of time-dependent discrimination, calibration, Brier score, likelihood, and decision-curve net benefit.

Existing genomic classifiers should not be considered interchangeable. Prolaris is primarily a cell-cycle progression classifier; Oncotype DX Genomic Prostate Score emphasises proliferation, androgen signalling, cellular organisation, and stromal response; whereas Decipher is principally oriented toward metastatic and prostate-cancer-specific outcomes [56]. Whether the proposed protein panel provides information independent of or incremental to these assays remains unknown.

Decipher is a 22-gene RNA classifier evaluated in formalin-fixed prostate tissue. In the NRG/RTOG 0126 analysis, pretreatment biopsies from 215 men receiving radiotherapy without ADT provided prognostic information for progression, biochemical failure, distant metastasis and prostate-cancer-specific mortality over a median follow-up of 12.8 years. The reported interaction with radiation dose was exploratory and should be distinguished from established treatment-predictive utility [57].

Prolaris is based on a cell-cycle progression signature reflecting proliferative activity. In a study of 141 men treated with definitive external-beam radiotherapy, the biopsy-derived 31-gene CCP score was associated with biochemical recurrence after adjustment for Gleason score, PSA, positive-core percentage and ADT. This supports prognostic assessment within a radiotherapy-treated population rather than demonstrating differential benefit from a treatment strategy [58].

The 17-gene Genomic Prostate Score assesses androgen signalling, proliferation, cellular organisation and stromal response in diagnostic biopsy tissue. In a retrospective cohort of 450 men treated predominantly with combined external-beam radiotherapy and brachytherapy, GPS was associated with biochemical failure and longer-term clinical outcomes. These findings support prognostic validity in that setting but do not establish a biomarker-by-treatment interaction [59].

Treatment-specific predictive analyses should be conducted only when the dataset contains an appropriate treatment contrast. Fractionation prediction requires comparative radiotherapy schedules, ADT prediction requires radiotherapy with versus without ADT or different ADT durations, and prediction of radiotherapy benefit relative to another treatment requires both treatment strategies. Formal biomarker-by-treatment interaction testing is essential. Interactions estimated from non-randomised comparisons should be considered exploratory because of confounding by indication and residual treatment-selection bias.

Clinical reports should follow REMARK principles, including transparent cohort assembly, assay methods, missing-data handling, cut-point selection, multivariable modelling, endpoint definitions, and complete follow-up [60]. External validation is required before clinical implementation. A four-antibody assay would be justified only if it demonstrated reproducible incremental value beyond standard clinical variables and relevant genomic classifiers while maintaining acceptable tissue requirements, interpretability, cost, and turnaround time.

The predominantly retrospective evidence and limited external validation currently restrict the clinical applicability of the panel. Retrospective transcriptomic and tissue studies remain vulnerable to selection bias, confounding by disease stage and treatment exposure, heterogeneous endpoints, sampling variation, and outcome-driven cut-point selection. Functional studies in cell lines or xenografts support mechanistic plausibility but do not establish clinical validity or transportability to pretreatment localised prostate cancer.

Among the four candidates, POSTN currently has the strongest tissue-level rationale, whereas MAPK8IP2, CDCA3, and NCAPG2 remain supported mainly by transcriptomic, functional, or limited tissue evidence, with incomplete independent protein-level validation. Spatial heterogeneity may be biologically informative, but it may also introduce measurement error; its incremental prognostic and clinical value should therefore be assessed empirically rather than assumed.

## 10. Conclusions

The four candidate proteins were selected to represent potentially complementary biological dimensions involving reactive stroma, stress signalling, checkpoint regulation, chromosome organisation, and tumour-cell persistence. They should not be interpreted as components of an established molecular axis or as a validated radiotherapy-predictive panel.

The heterogeneity of the evidence base limits direct comparison across studies. A marker associated with outcome in metastatic or previously treated disease may reflect treatment history, disease burden, or lineage evolution rather than the same biological process operating in untreated localised prostate cancer. Likewise, functional effects in cell lines or xenografts support mechanistic plausibility but do not establish the clinical validity of protein-level measurements or radiotherapy-specific predictive value.

Their combined assessment is biologically plausible as a hypothesis-generating strategy; however, functional connectivity, spatial co-occurrence, incremental prognostic value, and radiotherapy-specific predictive value remain unproven.

Among the four candidates, POSTN currently has the strongest tissue-level rationale, whereas MAPK8IP2, CDCA3, and NCAPG2 remain supported mainly by transcriptomic, functional, or limited tissue evidence, with incomplete independent protein-level validation. None of the four proteins has established predictive value for radiotherapy, fractionation, androgen-deprivation therapy (ADT), or treatment selection.

At present, the predominantly retrospective evidence and limited external validation restrict the clinical applicability of this candidate framework. The proposed markers should therefore be considered candidates for further investigation rather than clinically actionable biomarkers. The next step is sequential analytical validation, patient-level model development, internal validation, algorithm locking, independent external validation, and comparison with established clinical and genomic classifiers. Only after these steps can the framework’s prognostic value, clinical utility, and potential treatment-specific predictive value be determined.

## Figures and Tables

**Figure 1:**
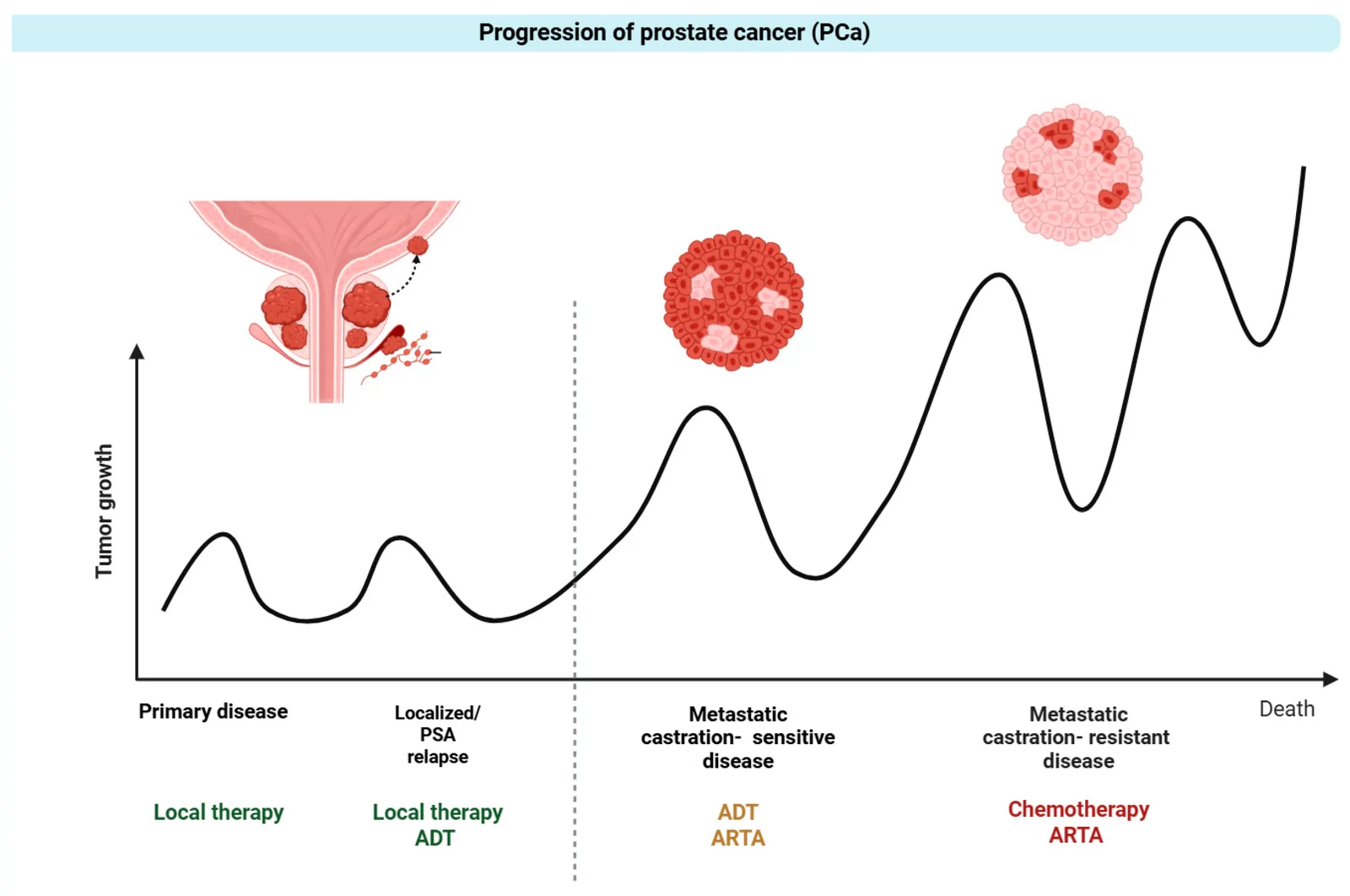
Clinical progression and treatment contexts in prostate cancer. Conceptual schematic showing the broad clinical trajectory from primary localised disease through biochemical recurrence, metastatic castration-sensitive prostate cancer, metastatic castration-resistant prostate cancer, and death. The principal treatment contexts shown are definitive local therapy; local therapy with androgen-deprivation therapy; androgen-deprivation therapy with androgen-receptor pathway-targeted agents; and chemotherapy with androgen-receptor pathway-targeted agents. This schematic illustrates major clinical states and treatment settings and does not represent a universal or strictly linear disease course. Created with BioRender.com.

**Figure 2:**
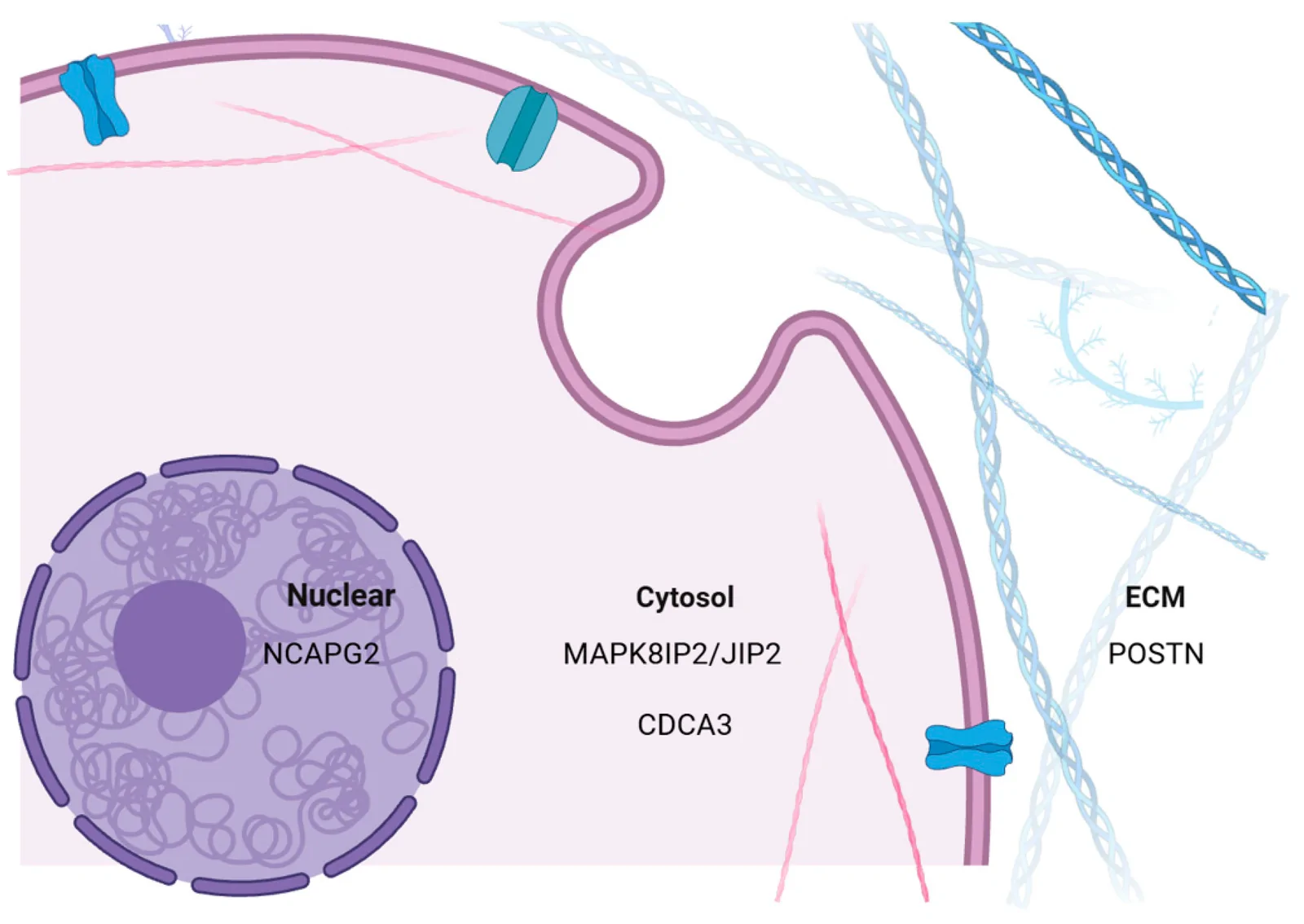
This figure illustrates the proposed biological compartments of emerging proteins periostin (POSTN), mitogen-activated protein kinase 8 interacting protein 2 (MAPK8IP2/JIP2), cell division cycle-associated protein 3 (CDCA3) and non-SMC condensin II complex subunit G2 (NCAPG2). Created with BioRender.com.

**Figure 3:**
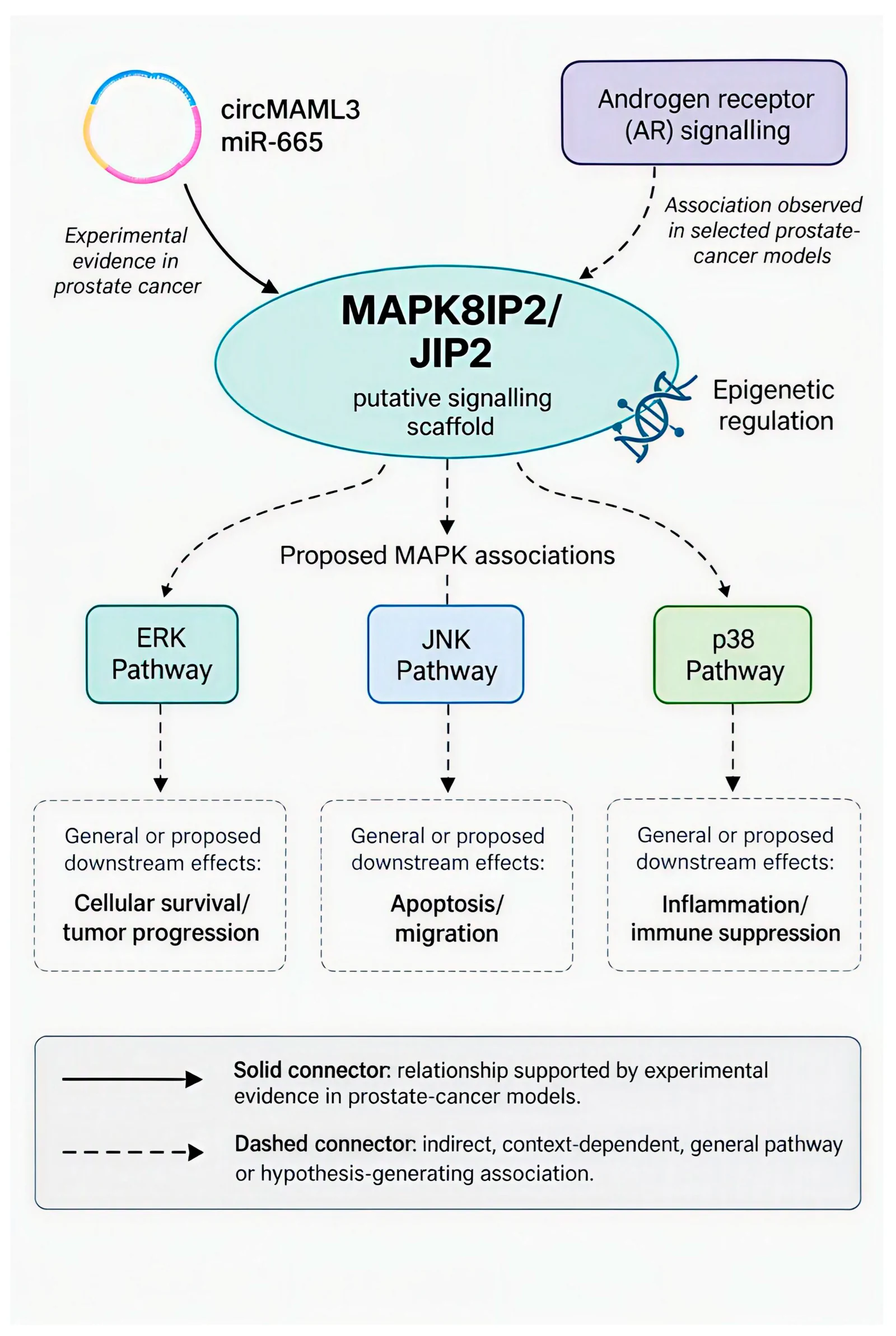
Experimental evidence and proposed links involving MAPK8IP2/JIP2 in prostate cancer. The solid connector represents the experimentally supported circMAML3/miR-665 axis. circMAML3 binds miR-665 and thereby relieves its repression of MAPK8IP2 expression. The dashed connector from androgen-receptor signalling indicates an association observed in selected prostate cancer models. The other dashed connectors indicate proposed links to the ERK, JNK and p38 pathways and their general or proposed downstream effects. Created with BioRender.com.

**Figure 4:**
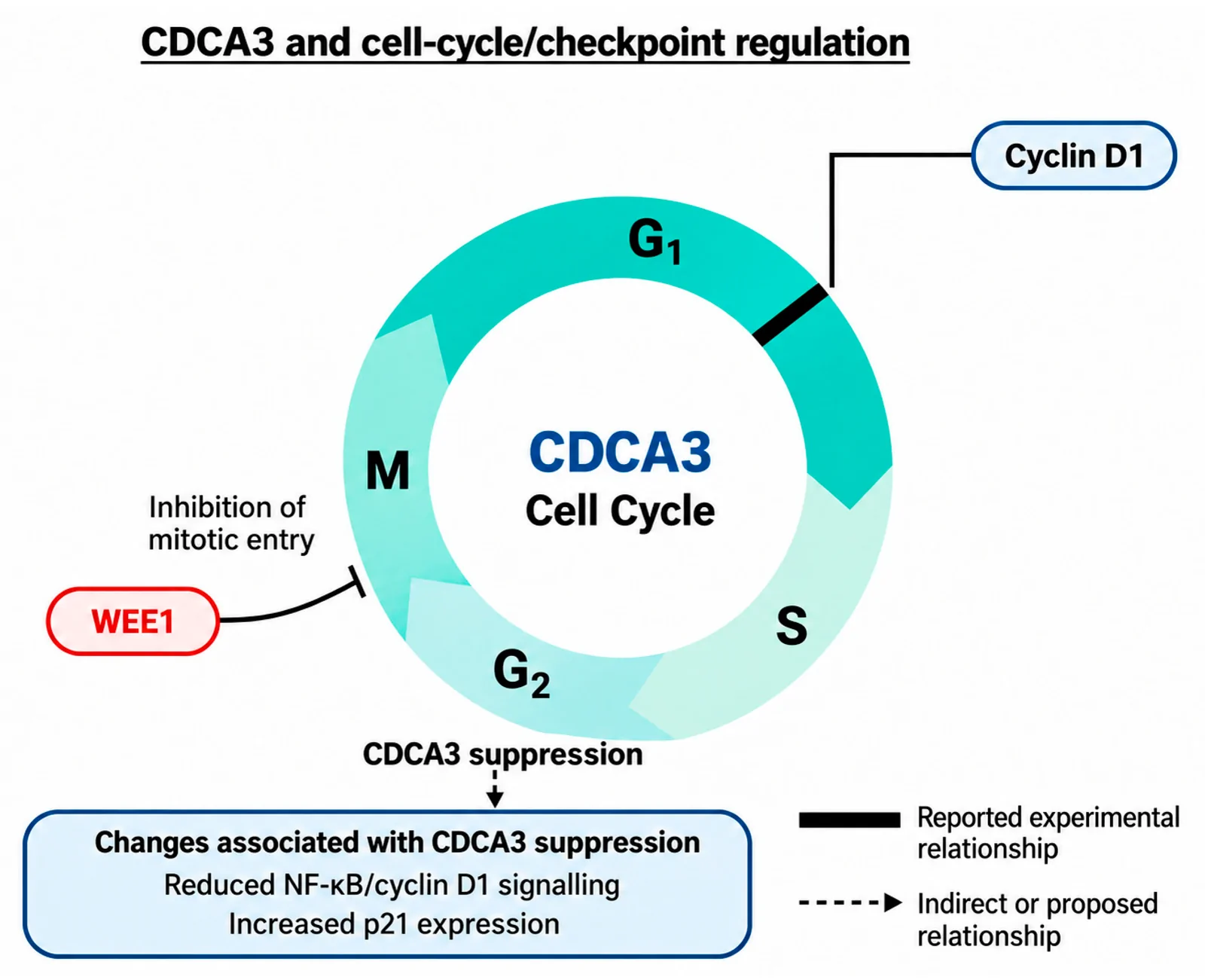
Proposed relationship between CDCA3 and cell-cycle/checkpoint regulation. CDCA3 is shown in the context of mitotic entry and cell-cycle progression, with WEE1 identified as a checkpoint regulator. The reported association between CDCA3 and WEE1-related checkpoint regulation is distinguished from the downstream changes in NF-κB/cyclin D1 signalling and p21 expression observed after CDCA3 suppression in prostate-cancer models. The latter are shown as indirect or pathway-associated effects and do not establish direct regulation of p21 by CDCA3. Solid arrows indicate relationships supported by experimental evidence; dashed arrows indicate indirect or proposed mechanisms. The figure does not establish that CDCA3 determines radiosensitivity or radioresistance. Created with BioRender.com.

**Table 1:** Biological, analytical and radiotherapy evidence profile of the candidate protein set.

Protein	Putative Dominant Biological Compartment	Principal Function/Pathway	Suggested IHC Readout	Current Prostate-Cancer Evidence	Direct Radiotherapy Evidence	Narrative Evidence Level for Prostate-Cancer Biological and Clinical Relevance
POSTN	Reactive stroma/ECM; sometimes tumour cells and osteoblasts	Integrin signalling, collagen organisation, tissue stiffness, invasion and metastatic niche	Stromal area/intensity; record epithelial staining separately	Transcriptomic and functional evidence: associations with stromal remodelling and aggressive disease. Protein-level prognostic evidence: recurrence associations in surgical tissue cohorts; independent assay and model validation remain incomplete.	None demonstrating predictive value; mechanistic plausibility only	Moderate—supported by multiple studies across complementary platforms, but compartment-specific and independent protein-level validation remain incomplete
MAPK8IP2/JIP2	Tumour-cell cytoplasm	JNK/p38-associated scaffold; stress adaptation; association with AR activity	Cytoplasmic intensity and percentage; consider phospho-JNK/p38 companion markers	Transcriptomic and functional evidence: public-cohort associations and experimental perturbation studies, with descriptive tissue IHC. Protein-level prognostic evidence: not established	No prostate radiotherapy experiments, irradiated clonogenic studies, or clinical biomarker-by-treatment interaction analyses	Limited–moderate—biologically plausible and functionally supported, but not yet established as a reproducible protein-level biomarker
CDCA3	Tumour-cell cytoplasm	Mitotic entry; checkpoint control; NF-kB/cyclin D1/p21 regulation	Tumour-cell H-score or intensity/percentage	Transcriptomic and functional evidence: transcript-level outcome associations and knockdown experiments. Protein-level prognostic evidence: not established; a separate IHC series showed associations with PSA, T stage and Gleason score	No direct evidence; plausible but directionally ambiguous	Limited—transcript-level outcome associations and protein-level clinicopathological correlations, without multivariable protein-level survival analysis or radiotherapy-specific validation
NCAPG2	Predominantly nuclear condensin biology; cytoplasmic signal also reported	Chromosome organisation and segregation; STAT3/c-MYC-associated stemness-related programmes	Separate nuclear and cytoplasmic scores	Transcriptomic and functional evidence: public-cohort analyses, perturbation experiments and xenografts. Protein-level prognostic evidence: tissue-expression findings without independently validated protein-level survival prediction	Co-expression analysis in an RT-plus-ADT dataset; no irradiation experiments or treatment-predictive validation	Limited—based on one principal study, without independent protein-level prognostic or radiotherapy-predictive validation

**Table 2:** Key prostate cancer studies informing the current evidence base.

Protein	Study	Cohort	Treatment Context	Data Type	Outcome	Follow-Up	Effect Estimate and 95% CI	Adjusted Covariates	External Validation	Dataset Provenance and Independence	Key Finding	Main Limitation
POSTN	Cattrini et al., 2020 [28]	48 patients with mCRPC, complemented by TCGA Firehose Legacy, MSKCC, SU2C/PCF and GEO analyses	mCRPC with heterogeneous prior systemic treatment; no radiotherapy-specific analysis	Public tumour transcriptomics; plasma POSTN dot-blot assay; exploratory CTC POSTN mRNA testing	Overall survival in the plasma cohort; disease-free survival in primary-tumour datasets	NR	Plasma POSTN > 10.4 versus ≤ 10.4 optical-density units: adjusted OS HR 3.49 (95% CI 1.18–10.36)	PSA, treatment line, number of metastatic sites, CTC status and AR-V7 status	No clearly independent clinical validation	TCGA and other public datasets are reused across publications; the plasma cohort is a separate exploratory series.	Circulating POSTN was associated with overall survival in mCRPC.	Small plasma cohort and outcome-selected threshold; circulating POSTN is not equivalent to compartment-specific tissue IHC
POSTN	Doldi et al., 2022 [29]	116 patients with prostate cancer assessed using radical prostatectomy specimens	Radical prostatectomy	Stromal POSTN immunohistochemistry with clinicopathological correlation	Biochemical recurrence-free survival; 31 biochemical recurrences	Median 84 months; range 65–104 months	Stromal POSTN score 2–3 versus 0–1: adjusted HR 5.75 (95% CI 1.73–19.17)	Resection-margin status was retained alongside periostin in the final multivariable model.	No independent external validation reported	Original 116-patient prostatectomy IHC cohort; fibroblast discovery reanalysed GSE76260	Supported stromal periostin as a candidate adjunct for risk assessment	Requires external validation, standardised scoring, and fixed thresholds
POSTN	Garcia-Marques et al., 2022 [30]	Discovery: 22 paired cancer/non-cancer specimens. Independent IHC/TMA series: 206 patients, including 53 with recurrence	Radical prostatectomy	LC–MS proteomics followed by POSTN/CALR/CTSD immunohistochemistry	Biochemical recurrence after surgery	Discovery: mean 6.8 years; TMA series: median 9 years	POSTN IHC score: Cox HR 1.60 (95% CI 1.04–2.48)	Three-protein Cox model including POSTN, CALR and CTSD; not a clinically adjusted survival model	Independent IHC/TMA series within the study; no subsequent external replication or radiotherapy validation	Original surgical proteomics and tissue cohorts; proteomics deposited as PXD028118	Proteomic discovery was followed by tissue-level confirmation of recurrence associations.	The staining/outcome comparison included 124 evaluable cases; the survival-model denominator was not clearly specified.
POSTN	Sun et al., 2022 [31]	TCGA analysis: 493 tumours; GEPIA DFS analysis: 492 patients; integrin-targeted PET cohort: 21 patients; cell and mouse models	Non-irradiated experimental models; clinical treatment exposure not specified for the public-data analyses	Public transcriptomics, POSTN perturbation experiments and integrin αvβ3-targeted PET	Public-cohort disease-free survival; experimental bone-metastatic burden and exploratory PET uptake	Median clinical follow-up NR	Unadjusted DFS HR, high versus low POSTN mRNA: 1.60; numerical 95% CI NR	None reported for the expression-stratified GEPIA survival analysis	No independent localised-radiotherapy validation	TCGA-derived survival analyses; GEPIA does not constitute an independent patient cohort	Tumour- and osteoblast-derived POSTN supported bone-metastasis-associated phenotypes.	Integrin-targeted PET uptake is not a direct measurement of POSTN protein; no radiotherapy-specific validation
MAPK8IP2	Zeng et al., 2022 [37]	TCGA: 499 cancers/52 normal samples; GSE70768: 125 cancers/73 normal samples, as reported; prostate-cancer cell models	Primarily untreated or retrospectively annotated cohorts; radiotherapy context NR	Transcriptomic analysis and in-vitro perturbation	TCGA progression-free interval and overall survival; experimental proliferation, migration and invasion	Median clinical follow-up NR	High versus low MAPK8IP2 mRNA: adjusted PFI HR 1.690 (1.045–2.733); adjusted OS HR 11.964 (1.184–120.873)	PFI: T stage, N stage, Gleason score, PSA, primary therapy outcome and residual tumour. OS: M stage, Gleason score, PSA and primary therapy outcome	Additional expression analyses; no independent protein-level prognostic validation	TCGA and SU2C/PCF datasets overlap with other MAPK8IP2 publications	High MAPK8IP2 was associated with adverse features and shorter outcomes; knockdown reduced malignant phenotypes.	Retrospective public datasets; no independent IHC or radiotherapy validation
MAPK8IP2	Huang et al., 2023 [16]	TCGA: 499 cancers; DKFZ: 116 patients, 105 with recurrence information; MSKCC/SU2C datasets; TMA containing 140 benign/malignant tissue sections	Surgical primary disease and metastatic castration-resistant disease; androgen/AR-pathway manipulation in cells; no radiotherapy-specific analysis	Transcriptomics, tissue-microarray IHC and functional cell assays	Transcript-level overall survival, disease-specific survival, progression-free interval and biochemical recurrence; tissue staining and AR-associated expression	NR	Reported Cox HR 1.519 (1.233–1.872); endpoint and model reporting are inconsistent	T stage, N stage, PSA, Gleason score and residual tumour are listed; simultaneous adjustment is unclear	Additional transcriptomic cohorts and descriptive IHC; no independent protein-level prognostic validation	TCGA/SU2C reuse across publications; cellular-expression datasets are not independent clinical prognostic cohorts	MAPK8IP2 was associated with adverse disease and androgen-receptor signalling activity.	Limited evidence across treatment-resistant states; no radiotherapy validation
MAPK8IP2/JIP2	Zhenhao et al., 2023 [36]	20 paired prostatectomy specimens; MAPK8IP2 qRT-PCR comparison in 16 pairs; TCGA: 497 cancers/52 normal samples; cell lines and xenografts	Prostatectomy without preoperative antitumour treatment; non-irradiated experimental models	circRNA/miRNA/mRNA assays, interaction/rescue experiments, Western blotting and xenograft IHC	Tissue expression, proliferation, migration, invasion and xenograft growth; exploratory TCGA survival association	NR	No adjusted clinical HR reported	No multivariable clinical prognostic model reported	Separate tissue-expression experiments; no independent clinical prognostic validation	TCGA is reused across publications; local tissue experiments are distinct	The proposed circMAML3–miR-665–MAPK8IP2 pathway was associated with malignant phenotypes.	Mechanistic and functional evidence; no radiotherapy assessment or independent clinical replication
CDCA3	Gu et al., 2022—functional study [17]	GSE27616: 9 cancers/4 normal samples; GSE3325: 13 cancers/6 normal samples; TCGA: 497 cancers/52 normal samples; 7 paired prostatectomy specimens; DU145 and PC-3 cells	Prostatectomy tissue collected without prior ADT, chemotherapy or radiotherapy; non-irradiated cell experiments	Public transcriptomics, GEPIA survival analysis, Western blotting and cell-line perturbation	Disease-free survival in GEPIA; tissue expression, proliferation, apoptosis and cell-cycle arrest	Median clinical follow-up NR	GEPIA disease-free survival association: *p* < 0.001; no covariate-adjusted HR reported	No multivariable prognostic model reported	No external clinical validation	TCGA-derived analyses reuse public patient data; additional expression datasets were GSE27616 and GSE3325	CDCA3 depletion induced cell-cycle arrest and apoptosis, with changes in NF-κB/cyclin D1/p21 signalling.	Small tissue series; no in vivo or radiotherapy experiments
CDCA3	Gu et al., 2023 [40]	TCGA transcriptomic cohort: 497 prostate cancers; separate IHC series: 60 prostate cancers and 20 normal samples	Retrospective TCGA analysis and a separate tissue series; no radiotherapy-specific outcome analysis	TCGA transcript analysis and IHC	TCGA: progression-free interval. IHC: associations with PSA, T stage and Gleason score	NR	TCGA, high versus low CDCA3 mRNA: univariable HR 2.237 (95% CI 1.447–3.458)	None; univariable analysis	No independent protein-level external validation	TCGA overlaps with other TCGA-based publications. The separate IHC series provides clinicopathological associations, not independent survival validation.	Higher CDCA3 mRNA was associated with poorer outcome in univariate analysis; protein expression was associated with PSA, T stage and Gleason score.	Univariate transcript-level analysis does not establish prognostic independence; IHC findings do not validate independent protein-level survival prediction.
NCAPG2	Zhang et al., 2024 [18]	TCGA: 497 cancers; IHC: 93 cancers/43 adjacent samples. Additional datasets: DKFZ2018, 292; GSE70769, 94; MSKCC2010, 218; GSE116918, 248. Dataset totals may differ from final survival-analysis denominators.	Includes GSE116918, treated with radiotherapy plus ADT, for co-expression analysis	Transcriptomic/proteomic analysis, TMA/IHC, functional perturbation, and xenografts	TCGA PFI; biochemical recurrence in additional transcriptomic cohorts; tissue expression and experimental malignant/stemness phenotypes	Median follow-up NR; survival analyses excluded follow-up shorter than 30 days	PFI: HR 1.65 (1.09–2.50). BCR: DKFZ2018, 5.8 (1.3–25); GSE70769, 5.4 (2.1–14); MSKCC2010, 5.5 (2.2–14)	No multivariable adjustment reported	Transcript-level recurrence associations across additional cohorts; no independent protein-level prognostic validation	Named public cohorts are reused across studies; GSE116918 supported co-expression analysis.	NCAPG2 promoted malignant and stemness-related phenotypes through STAT3/c-MYC signalling.	No treatment-interaction analysis or independent protein-level prognostic validation
POSTN/stroma	Okano et al., 2024 [32]	103 MRI-suspected extraprostatic-extension lesions from 96 radical-prostatectomy patients	Preoperative MRI with prostatectomy histopathology as the reference	Multiparametric MRI, histopathology and stromal POSTN immunohistochemistry	MRI–pathology discordance for extraprostatic extension	NA for the imaging–pathology comparison	Stromal POSTN expression: adjusted OR for pEPE 0.74 (95% CI 0.62–0.87), as reported in Figure 3; the main text reports 0.73	Grade Group, intraductal carcinoma, anterior versus posterior tumour location, and capsular contact length	None; single-centre retrospective cohort without an independent validation set	Independent imaging–pathology cohort; POSTN-specific dataset overlap not established	Stromal POSTN was associated with MRI–pathology discordance.	Selected MRI-positive surgical cohort; no survival or radiotherapy prediction. POSTN effect coding is not fully clear, and text and figure estimates differ.

Cohort size, treatment context, assay type, endpoint, follow-up, effect estimates, adjusted covariates, and external validation status are reported when available in the original publication. “Not reported” indicates that the relevant information was not available in the source article. Analyses using the same public dataset are not considered independent clinical replication. When dataset independence could not be established, overlap status is reported as unclear. Transcript-level findings and protein-level tissue findings are presented as distinct evidence categories. Information that could not be verified from the accessible source is identified separately and is not classified as “not reported”. *Abbreviations**:*** AR, androgen receptor; CI, confidence interval; GEO, Gene Expression Omnibus; HR, hazard ratio; IHC, immunohistochemistry; NA, not applicable; NR, not reported; PCa, prostate cancer; TCGA, The Cancer Genome Atlas; TMA, tissue microarray. OR, odds ratio; pEPE, pathological extraprostatic extension.

**Table 3:** Testable radiobiological hypotheses and proposed validation strategies.

Marker	Direct Prostate-Cancer Evidence Without Radiotherapy	Direct Irradiation Evidence in Prostate Cancer	Indirect Evidence and Biological Rationale	Potential Radiobiological Hypothesis	Potential Predictive Hypothesis	Key Uncertainty	Priority Experiment/Clinical Analysis
POSTN	Stromal POSTN has been associated with aggressive disease, recurrence-related phenotypes, extracellular-matrix remodelling, and bone-metastatic biology in non-irradiated or advanced prostate cancer. Evidence is compartment-dependent and includes transcriptomic, proteomic, tissue, and experimental studies.	No study has demonstrated that pretreatment POSTN modifies clonogenic survival, radiation-induced DNA-damage repair, in vivo radiotherapy response, or the relative benefit of a radiotherapy regimen in prostate cancer.	General radiobiological literature supports possible roles for cancer-associated fibroblasts, extracellular-matrix remodelling, hypoxia, integrin signalling, and stromal survival. These data are indirect and do not establish POSTN-specific radiation resistance.	POSTN-rich cancer-associated fibroblast (CAF) stroma and extracellular-matrix remodelling may be associated with hypoxia, integrin-mediated survival, residual-disease niches, and increased recurrence or metastatic risk under standard treatment.	POSTN abundance may modify the relative benefit of radiotherapy dose, fractionation, or a hypoxia-/stroma-directed intervention. This remains unproven.	Prognostic stromal aggressiveness may be incorrectly interpreted as radiation-specific resistance.	Compartment-specific pretreatment IHC; hypoxia profiling and clonogenic assays for mechanistic support. In a clinical cohort, estimate the prespecified POSTN × treatment interaction for biochemical recurrence or metastasis-free survival.
MAPK8IP2	Transcriptomic and functional prostate-cancer studies associate MAPK8IP2 with adverse disease features, stress-signalling programmes, androgen-receptor-related biology, and malignant cellular phenotypes. Protein-level and spatial validation remain limited.	No prostate-cancer study has evaluated MAPK8IP2 in irradiated models, radiation-induced DNA-damage repair, clonogenic survival, or a clinical biomarker-by-radiotherapy interaction.	General JNK/p38 stress-signalling biology provides an indirect rationale because these pathways may mediate apoptosis or adaptation after cellular stress. This does not establish MAPK8IP2 as a radiation regulator.	MAPK8IP2 expression may mark a tumour-cell state associated with stress signalling, androgen-receptor-related biology, and recurrence risk within a defined treatment context.	MAPK8IP2 may modify the relative effect of fractionated radiotherapy or a pathway-directed radiosensitising strategy through altered JNK/p38 stress-response kinetics.	JNK/p38 signalling may promote apoptosis or adaptive recovery; an association with outcome does not establish radiation-specific treatment modification.	AR-defined models with MAPK8IP2 perturbation, non-irradiated control versus clinically relevant fractionated irradiation, clonogenic survival, and phospho-pathway readouts. In a clinical cohort, test the prespecified MAPK8IP2 × treatment interaction rather than comparing outcomes only within marker strata.
CDCA3	Functional, transcriptomic, and limited tissue studies associate CDCA3 with mitotic entry, checkpoint dysregulation, proliferation, and adverse clinicopathological features. Transcript-level outcome associations and protein-level clinicopathological correlations should be interpreted separately.	No direct prostate-cancer evidence demonstrates altered clonogenic survival, radiation-induced DNA-damage repair, mitotic catastrophe, or radiotherapy-specific treatment interaction after CDCA3 perturbation.	General checkpoint and mitotic-catastrophe literature supports a possible relationship between cell-cycle control and radiation response. Evidence from other tumour types is indirect and cannot establish CDCA3-specific effects in prostate cancer.	Checkpoint dysregulation may be associated with accelerated repopulation or mitotic catastrophe and therefore with recurrence or poor outcome.	CDCA3 status may modify the effect of radiotherapy or of radiotherapy combined with a WEE1-directed strategy.	Poor prognosis does not imply radioresistance; direction may depend on TP53/WEE1 status and other cell-cycle features.	Assess clonogenic survival, mitotic catastrophe, and treatment response after CDCA3 perturbation. Clinical predictive analysis requires a comparative radiotherapy or radiosensitiser cohort with a prespecified CDCA3 x treatment interaction.
NCAPG2	One principal multi-platform prostate-cancer study supports associations with malignancy, chromosome organisation, STAT3/c-MYC signalling, and stemness-related phenotypes using public cohorts, tissue material, proteomics, functional assays, and xenografts. Independent protein-level replication is lacking.	No direct irradiation experiments or clinical treatment-interaction analyses. An RT-plus-ADT dataset was used for NCAPG2–MYC co-expression analysis.	General literature on stemness, chromosome maintenance, genomic instability, and DNA-damage response provides indirect biological plausibility. These data do not establish NCAPG2-specific radioresistance.	Condensin activity and STAT3/c-MYC-associated stemness may identify tumours with increased recurrence risk or post-treatment repopulation under standard therapy.	NCAPG2 may modify the relative benefit of radiotherapy or of radiotherapy combined with a DNA-damage-response intervention.	The same dependency that marks aggressive biology may create a therapeutic vulnerability; “high risk” and “radiation resistant” are not equivalent.	Combine NCAPG2 perturbation with radiation and assess DNA repair, chromosome aberrations, and tumoursphere formation. In clinical cohorts, test whether NCAPG2 changes the treatment effect rather than merely predicting outcome after radiotherapy.

## Data Availability

No new data were created or analyzed in this study. Data sharing is not applicable to this article.

## Supplementary Materials

The following supporting information can be downloaded at: https://www.mdpi.com/article/10.3390/biology15212026/s1, The Supplementary Methods provide the marker-specific PubMed search strategies, retrieval counts, screening results and details of the four additional publications.

## Abbreviations

The following abbreviations are used in this manuscript:

ADT	Androgen deprivation therapy
AKT	Protein kinase B
AP1	Activator protein 1
AR	Androgen receptor
ATF2	Activating transcription factor 2
CAFs	Cancer-associated fibroblasts
CDC42	Cell division cycle 42
CDCA	Cell division cycle associated
CDCA3	Cell division cycle-associated protein 3
CDK12	Cyclin-dependent kinase 12
circMAML3	Circular RNA derived from the MAML3 gene
c-MYC	MYC proto-oncogene protein
DNA	Deoxyribonucleic acid
ECM	Extracellular matrix
ERK	Extracellular signal-regulated kinase
EZH2	Enhancer of zeste homolog 2
FAK	Focal adhesion kinase
GEO	Gene Expression Omnibus
IF	Immunofluorescence
IHC	Immunohistochemistry
ISUP	International Society of Urological Pathology
JIP2	JNK-interacting protein 2
JNK	c-Jun N-terminal kinase
m^6^A	N^6^-methyladenosine
MAPK	Mitogen-activated protein kinase
MAPK8IP2	Mitogen-activated protein kinase 8 interacting protein 2
miR-665	MicroRNA-665
MRI	Magnetic resonance imaging
NCAPG2	Non-SMC condensin II complex subunit G2
NEPC	Neuroendocrine prostate cancer
NF-κB	Nuclear factor kappa B
PCa	Prostate cancer
PET	Positron emission tomography
PI3K	Phosphoinositide 3-kinase
PIA	Proliferative inflammatory atrophy
PIN	Prostatic intraepithelial neoplasia
POSTN	Periostin
PSA	Prostate-specific antigen
PTEN	Phosphatase and tensin homolog
RAC	Ras-related C3 botulinum toxin substrate
RAS	Rat sarcoma
RB1	Retinoblastoma 1
REMARK	Reporting Recommendations for Tumor Marker Prognostic Studies
RHO	Ras homolog
SANRA	Scale for the Assessment of Narrative Review Articles
SMC	Structural maintenance of chromosomes
SRF	Serum response factor
STAT3	Signal transducer and activator of transcription 3
TCF*	Ternary complex factor
TCGA	The Cancer Genome Atlas
TOME-1	Trigger of mitotic entry 1
TP53	Tumor protein p53
WEE1	WEE1 G2 checkpoint kinase
