# Supplementary material for: Stroma, Stress and Mitotic Control in Prostate Cancer: Evaluating Periostin, MAPK8IP2, CDCA3 and NCAPG2 Within an Evidence Gap Framework
Source: Biology (Basel). 2026 Nov 7;15(21):2026. doi: 10.3390/biology15212026 (PMC13761424; doi:10.3390/biology15212026)
Supplement: Supplementary file 1 [file biology-15-02026-s001.zip › biology-4623210-supplementary.pdf]

Review

Stroma, stress and mitotic control in prostate cancer: evaluating periostin, MAPK8IP2, CDCA3 and NCAPG2 within an evidence gap framework

Supplementary Materials

We ran the core PubMed/MEDLINE query and the four marker-specific queries on 25 October 2026. All five used the same prostate cancer terms and covered publications dated from 1 January 2021 through 14 July 2026.

The core query retrieved 24 records. The four marker-specific queries returned 19 records in total, all of which were also captured by the core query. The five queries therefore yielded 43 retrievals and 24 unique records. We excluded three records after reviewing their titles and abstracts and assessed the remaining 21 articles in full. We excluded nine articles because they concerned postoperative or metastatic disease and provided no tissue biomarker, mechanistic or radiotherapy evidence relevant to the review. We retained 12 publications from the date-restricted search.

Targeted PubMed searches without a lower publication-date limit and citation tracking identified four earlier publications, bringing the total to 16. Cattrini et al. (2020, PMID 32416542) examined periostin in primary and advanced prostate cancer. Wang and Ouyang (2012, PMID 22305559) discussed periostin in the metastatic niche. Wagner and Nebreda (2009, PMID 19629069) reviewed JNK and p38 signalling in cancer. Ono et al. (2003, PMID 14532007) investigated the contributions of condensins I and II to mitotic chromosome architecture.

The four marker-specific search strings and their retrieval counts are listed below.

POSTN + additional terms

(("Prostatic Neoplasms"[Mesh] OR "prostate cancer"[Title/Abstract] OR "prostatic neoplasms"[Title/Abstract] OR "prostate adenocarcinoma"[Title/Abstract]) AND (POSTN[Title/Abstract] OR periostin[Title/Abstract]) AND (radiotherap*[Title/Abstract] OR radiation[Title/Abstract] OR radioresistan*[Title/Abstract] OR "biochemical recurrence"[Title/Abstract] OR prognos*[Title/Abstract] OR immunohistochem*[Title/Abstract] OR "androgen receptor"[Title/Abstract] OR "cancer stem cell"[Title/Abstract] OR "extracellular matrix"[Title/Abstract] OR JNK[Title/Abstract] OR p38[Title/Abstract] OR STAT3[Title/Abstract] OR MYC[Title/Abstract] OR WEE1[Title/Abstract] OR "cell cycle"[Title/Abstract]) AND ("2021/01/01"[Date - Publication] : "2026/07/14"[Date - Publication]))

Records retrieved: 8.

MAPK8IP2/JIP2 + additional terms

(("Prostatic Neoplasms"[Mesh] OR "prostate cancer"[Title/Abstract] OR "prostatic neoplasms"[Title/Abstract] OR "prostate adenocarcinoma"[Title/Abstract]) AND

(MAPK8IP2[Title/Abstract] OR JIP2[Title/Abstract]) AND (radiotherap*[Title/Abstract] OR radiation[Title/Abstract] OR radioresistan*[Title/Abstract] OR "biochemical recurrence"[Title/Abstract] OR prognos*[Title/Abstract] OR immunohistochem*[Title/Abstract] OR "androgen receptor"[Title/Abstract] OR "cancer stem cell"[Title/Abstract] OR "extracellular matrix"[Title/Abstract] OR JNK[Title/Abstract] OR p38[Title/Abstract] OR STAT3[Title/Abstract] OR MYC[Title/Abstract] OR WEE1[Title/Abstract] OR "cell cycle"[Title/Abstract]) AND ("2021/01/01"[Date - Publication] : "2026/07/14"[Date - Publication]))

Records retrieved: 3.

CDCA3/TOME-1 + additional terms

(("Prostatic Neoplasms"[Mesh] OR "prostate cancer"[Title/Abstract] OR "prostatic neoplasms"[Title/Abstract] OR "prostate adenocarcinoma"[Title/Abstract]) AND (CDCA3[Title/Abstract] OR "TOME-1"[Title/Abstract] OR TOME1[Title/Abstract]) AND (radiotherap*[Title/Abstract] OR radiation[Title/Abstract] OR radioresistan*[Title/Abstract] OR "biochemical recurrence"[Title/Abstract] OR prognos*[Title/Abstract] OR immunohistochem*[Title/Abstract] OR "androgen receptor"[Title/Abstract] OR "cancer stem cell"[Title/Abstract] OR "extracellular matrix"[Title/Abstract] OR JNK[Title/Abstract] OR p38[Title/Abstract] OR STAT3[Title/Abstract] OR MYC[Title/Abstract] OR WEE1[Title/Abstract] OR "cell cycle"[Title/Abstract]) AND ("2021/01/01"[Date - Publication] : "2026/07/14"[Date - Publication]))

Records retrieved: 3.

NCAPG2/condensin II + additional terms

(("Prostatic Neoplasms"[Mesh] OR "prostate cancer"[Title/Abstract] OR "prostatic neoplasms"[Title/Abstract] OR "prostate adenocarcinoma"[Title/Abstract]) AND (NCAPG2[Title/Abstract] OR "condensin II"[Title/Abstract]) AND (radiotherap*[Title/Abstract] OR radiation[Title/Abstract] OR radioresistan*[Title/Abstract] OR "biochemical recurrence"[Title/Abstract] OR prognos*[Title/Abstract] OR immunohistochem*[Title/Abstract] OR "androgen receptor"[Title/Abstract] OR "cancer stem cell"[Title/Abstract] OR "extracellular matrix"[Title/Abstract] OR JNK[Title/Abstract] OR p38[Title/Abstract] OR STAT3[Title/Abstract] OR MYC[Title/Abstract] OR WEE1[Title/Abstract] OR "cell cycle"[Title/Abstract]) AND ("2021/01/01"[Date - Publication] : "2026/07/14"[Date - Publication]))

Records retrieved: 5.
